# Editorial: Benefits of emotional intelligence in the educational and health sphere

**DOI:** 10.3389/fpsyg.2024.1359150

**Published:** 2024-02-21

**Authors:** Isabel Mercader Rubio, Nieves Gutiérrez Ángel, Sonia Brito-Costa, José Juan Carrión-Martínez

**Affiliations:** ^1^Department of Psychology, University of Almeria, Almería, Spain; ^2^Human Potential Development Center, Institute of Applied Research, Polytechnic Institute of Coimbra, Coimbra, Portugal; ^3^Department of Education, University of Almeria, Almería, Spain

**Keywords:** benefits, emotional intelligence, education, health, editorial

This editorial entitled Benefits of emotional intelligence in the field of education and health aims to address from a double perspective one of the most studied constructs in psychology today: emotional intelligence.

In this way, it contains studies related to the relationship that currently exists between emotional intelligence and education, whether formal or informal, and the educational processes implicit in it. And on the other hand, from a health perspective, it provides different studies that aim to strengthen the correspondence between emotional intelligence and physical, emotional and psychological wellbeing.

In its entirety, this editorial is made up of five manuscripts which analyze emotional intelligence from different types of population, however all of them share the fact that they are focused on the educational context.

However, we should also note that the manuscript by Hasan et al. examines the trait profiles of emotional intelligence in different professionals, such as bankers, doctors, engineers, policemen, lawyers or teachers in Kuwait and the relationship between this construct and job performance. Thus, it offers us a novel vision of emotional intelligence. Likewise, the main contributions of this work highlight the importance of emotional capacity at work.

Meanwhile, fully focused on the educational context, the manuscript presented by Kong et al. analyses emotional intelligence and its relationship with learning anxiety in a total of 470 adolescents in China. The main contributions of this manuscript coincide in pointing to emotional intelligence as a measure to reduce anxiety caused by different learning situations. And how it can become a way to avoid or alleviate such situations related to negative emotions for students.

In the same line of research is the manuscript by Tang and He, who in this case analyze the relationship between emotional intelligence and motivation in a total of 336 university students in China. The main findings of this manuscript indicate that different interventions aimed at developing emotional intelligence also improve self-efficacy, social support, motivation and academic performance of university students. Therefore, it offers a completely different view from that of the previous manuscript. That is, if the manuscript presented by Kong et al. indicates that once negative emotions exist, emotional intelligence can alleviate them. The manuscript submitted by Tang and He shows that one way to prevent these events is to work on the emotional intelligence of students. It also shows that nowadays it can even be considered a protective factor for relevant issues such as self-efficacy, social support, motivation and academic performance.

Similarly, Huang and Zeng analyze in this case the relationship between academic performance and emotional intelligence in a total of 5,703 students in China. The main contribution of this manuscript lies in the establishment of the existence of positive connections between both variables, and in the importance of influencing the development of emotional strategies and interventions in students in order to improve their academic performance. In other words, proving the direct and positive correlation between high levels of emotional intelligence and better academic performance.

Finally, also within the educational context but this time taking practicing educators as a sample, the manuscript presented by Floman et al. examines different dimensions of emotional intelligence, such as regulation and support. They conclude that investing in emotional training programmes for educators has a positive impact on their psychological wellbeing. Thus, it is demonstrated that emotional intelligence in the educational field should not be something exclusive to students, but also to teachers. And that it is an improvement in the psychological wellbeing of education professionals.

## Author contributions

IM: Writing—original draft, Writing—review & editing. NG: Writing—original draft, Writing—review & editing. SB-C: Writing—original draft, Writing—review & editing. JC-M: Writing—original draft, Writing—review & editing.

