# Supplementary material for: Editorial: Benefits of emotional intelligence in the educational and health sphere
Source: Front Psychol. 2024 Feb 21;15:1359150. doi: 10.3389/fpsyg.2024.1359150 (PMC10915243; doi:10.3389/fpsyg.2024.1359150)
Supplement: Supplementary file 1 [file Data_Sheet_1.PDF]

El editorial titulado Beneficios de la Inteligencia Emocional en el Ámbito Educativo y Sanitario está compuesto en su totalidad por cinco manuscritos, los cuales analizan la inteligencia emocional desde distintos tipos de población, sin embargo todos ellos comparten el hecho de estar centrados en el contexto educativo, salvo el manuscrito elaborado por Hassan et al (2023) quienes examinan los perfiles de rasgo de inteligencia emocional en distintos profesionales, como banqueros, médicos, ingenieros, policías, abogados o profesores de Kuwait y la relación entre dicho constructo y el desempeño laboral. Asimismo las principales aportaciones de este trabajo resaltan el hecho de la importancia de la capacidad emocional en el trabajo.

Por su parte, plenamente centrados en el el contexto educativo, encontramos el manuscrito presentado por Kong et al. (2023) analiza la inteligencia emocional y su relación con la ansiedad ante el aprendizaje en un total de 470 adolescentes de China. Las principales aportaciones de este manuscrito coinciden en señalar a la inteligencia emocional como una medida para reducir la ansiedad provocada por distintas situaciones de aprendizaje.

En esta misma línea de investigación se sitúa el manuscrito elaborado por Tang & He (2023), quienes en este caso analizan la relación entre la inteligencia emocional y la motivación en un total de 336 universitarios de China. Las principales hallazgos de este manuscrito indican que a partir de distintas intervenciones que tienen por finalidad desarrollar la inteligencia emocional también se mejora la autoeficacia, el apoyo social, la motivación y el rendimiento académico de los estudiantes universitarios.

De la misma manera, Huang & Zeng (2023) analizan en este caso la relación entre el rendimiento académico y la inteligencia emocional en un total de 5703 alumnos De China. La principal aportación de este manuscrito reside en el establecimiento de la existencia de conexiones positivas entre ambas variables, y en la importancia de incidir en el desarrollo de estrategias e intervenciones emocionales en los estudiantes con la finalidad de mejorar su rendimiento académico.

Finalmente, también dentro del contexto educativo pero en esta ocasión tomando como muestra a educadores en ejercicio, el manuscrito presentado por Floman et al. (2023) examina distintas dimensiones de la inteligencia emocional, como la regulación y el apoyo. Llegando a la conclusión de que invertir en programas de formación emocional en educadores repercute positivamente en el bienestar psicológico de los mismos.
